# Neurosyphilis masquerading as oculomotor nerve palsy in a healthy middle-aged man: Case report and review of the literature

**DOI:** 10.1016/j.idcr.2021.e01237

**Published:** 2021-07-24

**Authors:** Fares Antaki, Kenan Bachour, Milanne Trottier, Laurent Létourneau-Guillon, Jacinthe Rouleau

**Affiliations:** a Department of Ophthalmology, Centre Hospitalier de l'Université de Montréal (CHUM), Université de Montréal, Montréal, Québec, Canada; b Department of Radiology, Centre Hospitalier de l'Université de Montréal (CHUM), Université de Montréal, Montréal, Québec, Canada

**Keywords:** Oculomotor nerve palsy, Cranial nerve palsy, Neurosyphilis, Neurology, Ophthalmology, Sexually transmitted infections, CNS infections, Neuroradiology

## Abstract

Acquired isolated oculomotor nerve palsy (ONP) is a commonly encountered clinical entity in ophthalmology. While most cases are due to microvascular ischemia, the diagnosis of ONP requires careful evaluation for alternate life-threatening etiologies. We present a case of isolated complete pupil-involving ONP in a healthy 47-year-old man in whom aneurysmal compression was initially suspected. Investigations later revealed a diagnosis of neurosyphilis. Neurosyphilis is an extremely rare cause of isolated ONP and seldom reported in the literature. Timely recognition of this disease by ophthalmologists can help orient patients to the appropriate neurology and infectious disease services they need.

## Introduction

The diagnosis of acquired isolated oculomotor nerve palsy (ONP) in adults requires careful evaluation. Clinical presentations vary and can be summarized as: complete or incomplete palsy (varying degree muscle involvement and ptosis) with or without pupil involvement [1]. While most cases are ischemic, compression from an aneurysm remains the most feared etiology justifying the need for neuroimaging in most cases [2]. In this report, we present a case of isolated complete pupil-involving ONP in a healthy middle-aged man in whom aneurysmal compression was initially suspected. Investigations later revealed a diagnosis of neurosyphilis. Neurosyphilis is an extremely rare cause of isolated ONP and to date, only 6 cases have been reported in the literature. We describe the laboratory and neuroimaging findings of this rare clinical presentation and review the literature on the topic. We also discuss the pathophysiology of this entity and its differential diagnosis.

## Case report

A 47-year-old man presented to the clinic with two weeks of headaches, progressive diplopia and right eyelid ptosis. He had no other neurological symptoms and review of systems was negative. He was a 10 pack-year smoker but had no past medical history, prior medication use or allergies. He had no ocular history. His family history was non-contributory. His sexual history was significant for unprotected same-sex intercourse with multiple partners over the past 10 years but no history of sexually transmitted infections. His vision was 20/30 in the right eye and 20/20 in the left. Examination revealed 80 prism diopters of exotropia with significant hypotropia and severe elevation, adduction and depression deficits (no movements from primary position) in the right eye. Abduction and incyclotorsion were preserved. The right pupil was dilated and poorly reactive to light and accommodation. No relative afferent pupillary defect was seen. Examination of the remaining cranial nerves revealed no abnormalities. Anterior segment and fundus examination was normal bilaterally. Neurological examination did not reveal ataxia, tremor or hemiparesis. Right isolated complete pupil-involving ONP was diagnosed.

**Fig. 1:**
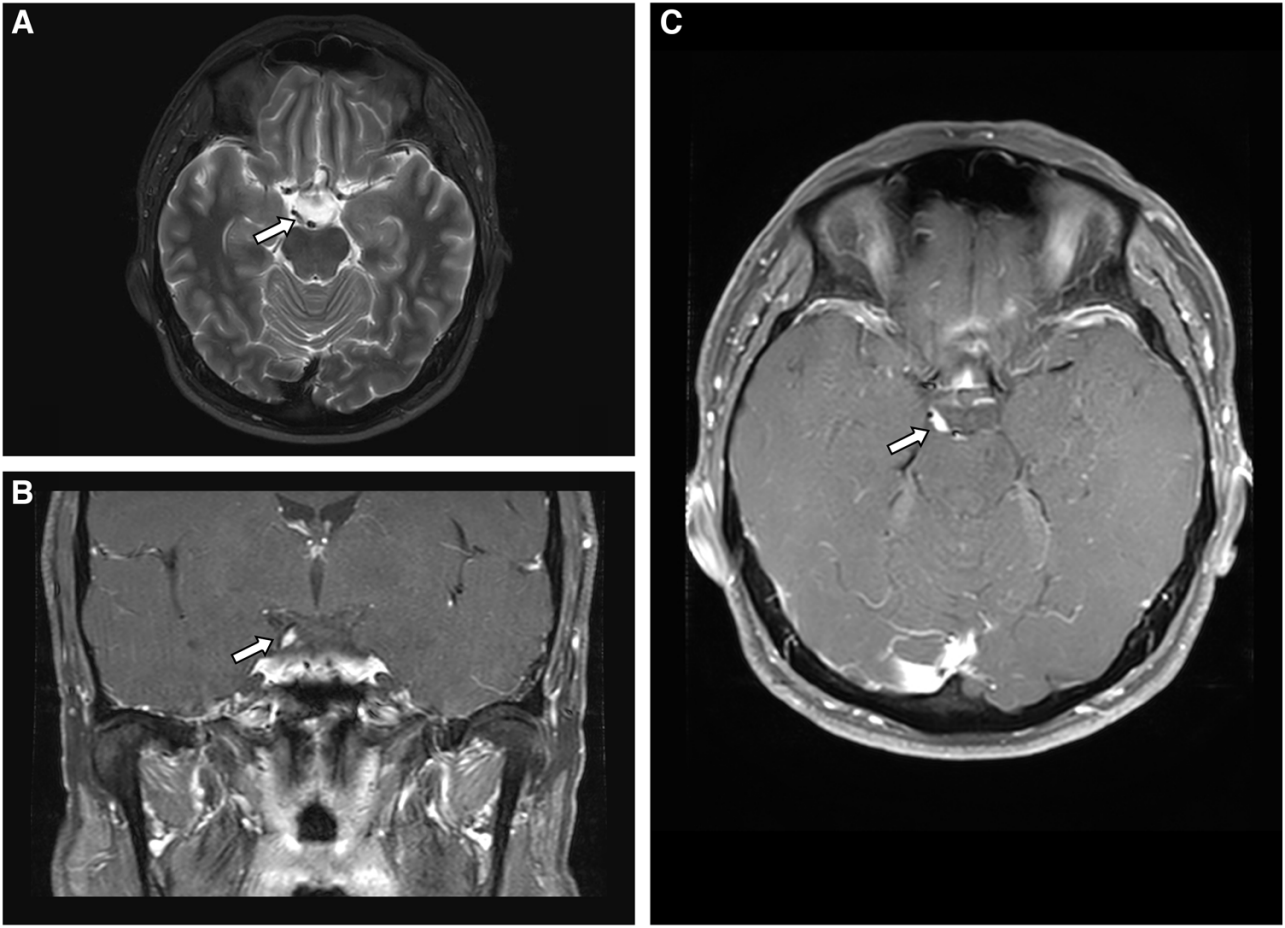
Axial T2-weighted (A) as well as coronal (B) and axial (C) T1-weighted MRI following administration of gadolinium showing thickening and enhancement of the right oculomotor nerve in its interpeduncular and suprasellar cisternal course.

Given the clinical presentation, aneurysmal compression was suspected and urgent brain computed tomography angiography (CTA) was obtained but came back normal. Brain magnetic resonance imaging (MRI) revealed thickening and enhancement of the right oculomotor nerve at the level of the interpeduncular and suprasellar cisterns (Fig. 1). There was no leptomeningeal enhancement nor other cranial nerve enhancement. Complete blood count, glycated hemoglobin, erythrocyte sedimentation rate and C-reactive protein serum levels were normal. Chest radiography did not reveal hilar adenopathy or signs of tuberculosis. The initial treponemal test (*Treponema pallidum* enzyme immunoassay) was reactive and was confirmed by a positive rapid plasma reagin test (at a 1:16 titer). Testing for human immunodeficiency virus (HIV) and tuberculosis (interferon gamma release assay, IGRA) was negative. Cerebrospinal fluid (CSF) analysis demonstrated elevated protein levels (0.83 g/L, normal: 0.15 – 0.40) and lymphocytic pleocytosis (30 × 10^6^/L, normal: < 5). A Venereal Disease Research Laboratory (VDRL) test performed on the CSF sample was reactive (at a 1:2 titer). Early neurosyphilis was diagnosed. The patient was treated with intravenous penicillin G, 4 million units every four hours administered at-home through a peripherally inserted central catheter for a total duration of 2 weeks. At the completion of treatment, symptomatic improvement was noted but the patient was lost to follow-up before a second ophthalmological assessment could be performed.

## Discussion

Neurosyphilis refers to the infection of the central nervous system by *T. pallidum* and may occur anytime during the course of the disease. Less than 10 % of untreated patients with syphilis go on to develop neurosyphilis, which can be classified into early forms and late forms [3]. In the early form, neurosyphilis involves the CSF, meninges, and vasculature. Patients are often neurologically asymptomatic. In the symptomatic phase, neurosyphilis can accompany features of secondary syphilis and patients may present with headache, stroke, meningitis, cranial nerve palsies, and hearing loss. In the later stages, the disease involves the brain and spinal cord parenchyma and manifests as dementia and tabes dorsalis [3].

In the literature, there are scarce reports of neurosyphilis presenting as isolated ONP. To our knowledge, only 6 cases supported by radiological findings have been reported in the English literature (Table 1) [[4], [5], [6], [7], [8], [9]]. Three of the 6 cases occurred in patients who were HIV positive [9,6,7]. HIV co-infection leads to faster disease progression from the asymptomatic to the symptomatic phase of neurosyphilis [2]. All patients improved with intravenous penicillin treatment but 4/6 had either radiological or clinical residual deficits during follow-up. Complete isolated unilateral, pupil-involving ONP was diagnosed in 5/6 cases. The authors did not consistently report results from angiographic studies performed to rule out aneurysmal compression.

**Table 1:** Summary of selected cases of neurosyphilis presenting as oculomotor nerve palsy, supported by magnetic resonance imaging (MRI) findings.

Authors	Year	Age, Sex	HIV	Palsy	Pupil-involving	MRI findings	Outcome	
							Clinical	Radiological
Vogl et al [4]	1993	44 F	–	Incomplete	Yes	Hyperintense lesion in the region of the exit of CN III from the brainstem in T2 (enhancing)	Resolved	Resolved
Seeley et al [5]	2004	54 M	–	Complete	Yes	Isointense lesion at the base of the midbrain tracing the course of CN III into the cavernous sinus in T1 and T2 (enhancing)	Partially resolved	Resolved
Corr et al [6]	2004	22 F	+	Complete OD Incomplete OS	Yes (OU)	Marked thickening of both CN III in T2 (enhancing)	Resolved	Resolved
Hess et al [7]	2013	39 M	+	Complete	Yes	Hyperintense enlargement of CN III in the interpeduncular cistern in T2-FLAIR (enhancing)	Partially resolved	Partially resolved
Park et al [8]	2015	43 M	–	Complete	Yes	Diffuse thickening of CN III in T2 (enhancing)	Partially resolved	Unknown
Silva et al [9]	2018	29 M	+	Complete	Yes	Diffuse thickening of the cisternal portion of CN III in T2 (enhancing)	Partially resolved	Resolved
Antaki et al	2020	47 M	–	Complete	Yes	Enlargement of CN III in the interpeduncular and suprasellar cisterns in T2 (enhancing)	Symptomatic improvement	Unknown

Abbreviations: HIV = human immunodeficiency virus; MRI = magnetic resonance imaging; F = female; M = male; Oculomotor nerve = third cranial nerve or CN III; T1 = T1-weighted images; T2 = T2-weighted images; FLAIR = Fluid-attenuated inversion recovery sequence; OD = right eye; OS = left eye; OU = both eyes. Enhancement refers to postgadolinium enhancement in T1-weighted images.

In the subarachnoid space, the oculomotor nerve courses inferior and lateral to the posterior communicating artery before entering the cavernous sinus, making it prone to damage from compression, infectious meningitis, infiltration and inflammation [5]. In our case, brain MRI showed enlargement of the oculomotor nerve with postgadolinium enhancement, potentially representing a nerve gumma from vigorous granulomatous response of the meninges. Other mechanisms by which neurosyphilis can cause ONP have been described: first, in the meningovascular phase of the disease, due to small vessel vasculitis and nerve infarction; and second, in granulomatous basal meningitis due to inflammation of the nerve [5]. Most MRI-supported cases in the literature have reported imaging findings similar to our case. The radiological differential diagnosis is broad. While the cisternal localisation, unilaterality and thickened appearance of the lesion were in favor of a schwannoma, this diagnosis was not retained initially given the rarity of this disease and the high clinical suspicion for an infectious process. Lyme disease and tuberculosis were considered but the absence of clinical context as well as other ancillary findings on neuroimaging made these etiologies unlikely. Neurosarcoidosis was also considered since cranial nerves may be involved either as part of leptomeningeal disease or in isolation. However, the absence of systemic evidence of sarcoidosis went against this diagnosis.

Our report is limited by the lack of long-term follow-up and clinical response to treatment. Despite that, we believe that the diagnosis of acquired isolated ONP due to neurosyphilis is strongly supported by the CSF findings and the radiological appearance that is in keeping with the literature. Our manuscript is important because it highlights the importance of obtaining an adequate sexual history as part of neurological history and examination. It also adds to the body of knowledge on the health disparities affecting Lesbian, Gay, Bisexual, and Transgender Health populations and men who have sex with men.

## Conclusion

Ophthalmologists, neurologists and infectious disease specialists should be aware of neurosyphilis as a potential cause of isolated ONP. This etiology is particularly important in young patients with at-risk sexual behaviors in the absence of aneurysmal compression. Timely recognition of this disease by ophthalmologists can help orient patients to the appropriate neurology and infectious disease services they need. Recognition of the MRI findings can also suggest this etiology in the appropriate clinical setting. CSF-VDRL must be obtained and testing for HIV co-infection should be considered.

## Patient consent

Informed consent was obtained from the patient for all procedures described in this case report. This report does not contain any personal identifying information.

## Funding

No funding was received for this study.

## Authorship

All authors attest that they meet the current ICMJE criteria for Authorship. F.A and J.R treated and followed the patient. F.A and K.B collected the data and performed the literature search. K.B drafted the initial manuscript. F.A prepared the figure. M.T and L.L.G interpreted the neuroradiology imaging. All authors had access to the underlying data which they verified. All authors edited and revised the manuscript before approving its final version.

## Declaration of Competing Interest

None of the authors has any financial/competing interests to disclose.
